# Multiple sclerosis presenting initially with a worsening of migraine symptoms

**DOI:** 10.1186/1129-2377-14-70

**Published:** 2013-08-09

**Authors:** Guan-Yu Lin, Chih-Wei Wang, Tsung-Ta Chiang, Giia-Sheun Peng, Fu-Chi Yang

**Affiliations:** 1 Department of Neurology, Tri-Service General Hospital, National Defense Medical Center, Taipei, Taiwan; 2 Department of Radiology, Tri-Service General Hospital, National Defense Medical Center, Taipei, Taiwan; 3 Department of Internal Medicine, Tri-Service General Hospital, National Defense Medical Center, Taipei, Taiwan

**Keywords:** Multiple sclerosis, Headache, Migraine, Periaqueductal gray matter

## Abstract

Multiple sclerosis (MS) is a chronic autoimmune disease that targets myelinated axons in the central nervous system. Headache has been reported as a subtle symptom of the onset of MS, with a variable frequency of 1.6–28.5%; however, it remains unclear whether headache is a true symptom of MS onset. Here, we report the case of a female patient who had a history of migraine without aura and experienced worsening of migraine-headache symptoms as the initial manifestation of MS. Three similar cases were reported previously; however, unlike this case, those cases had no history of migraine without aura. In our case, we excluded factors that could trigger migraine attacks, such as changes in weather, drugs, alcohol, caffeine withdrawal, stress, fatigue, lack of sleep, hormonal therapy, diet, and hunger. The patient had one episode of MS attack with the simultaneous presence of asymptomatic gadolinium-enhancing and non-enhancing lesions, including hyperintense lesions in the bilateral periventricular white matter, body of the corpus callosum, and periaqueductal grey matter, as observed on the T2-weighted images obtained at the first brain magnetic resonance imaging. In addition, after the injection of gadolinium contrast, ring enhancement over these lesions was noted in T1-weighted images, which was suggestive of active demyelination. MS was diagnosed according to the McDonald criteria (2010 revision). We conclude that MS with periaqueductal grey matter involvement may present with worsening migraine. It is important to be cautious if any secondary causes exist, especially when the patient has a history of migraine without aura. MS should be one of the differential diagnoses in young women showing a change in headache pattern or poor clinical drug response to migraine treatment accompanied by episodes of focal neurological deficit. Failure to recognize MS may lead to inappropriate treatment and worse prognosis; early diagnosis in patients with MS is essential to improve their clinical outcomes and quality of life.

## Background

Multiple sclerosis (MS) is a chronic autoimmune disease that affects the myelinated axons in the central nervous system (CNS) [1]. This disease affects women more frequently than men. The estimated female-to-male ratio of MS incidence increased from 1.4 in 1955 to 2.3 in 2000, according to a systematic review of 28 epidemiologic studies [2]. The average age of onset of MS is 30 years, and the disease starts approximately five years earlier in women than it does in men [3]. MS symptoms at presentation vary individually and are unpredictable [4]. Headache is not generally regarded as a symptom of MS, although it occurs in more than half of the cases of MS [5]. Whether headache is a symptom of MS onset remains an open question [5]. Recently, we encountered a woman with MS, as diagnosed according to the 2010 revisions of the McDonald criteria, whose initial presentation was worsening migraine [6]. After steroid therapy, the patient returned to the remission stage without obvious neurological sequel, and the headache improved significantly.

## Case presentation

A 33-year-old woman experienced severe right- or left-sided parietal–temporal throbbing headache accompanied by blurred vision, photophobia, vomiting, and anorexia at a frequency of 1–2 days per month since she was an adolescent. She was diagnosed with migraine without aura and received no prophylactic treatment. One month ago, she exhibited more severe, right-sided, parietal–temporal throbbing headaches characterized by a prolonged duration that increased in frequency to more than 15 days per month. She showed a poor medical response to sumatriptan and naproxen therapy. A few days after sumatriptan and naproxen therapy, she experienced the first MS episode of blurred vision over both the eyes and right facial numbness, which subsided quickly and spontaneously after 24 hours. Because the symptoms subsided quickly after 24 hours, the patient did not come to our outpatient department (OPD) at that time. No objective evidence of alteration of vision or facial sensation was observed. According to our detailed recordings of the patient’s history, she had never experienced these symptoms before.

**Figure 1:**
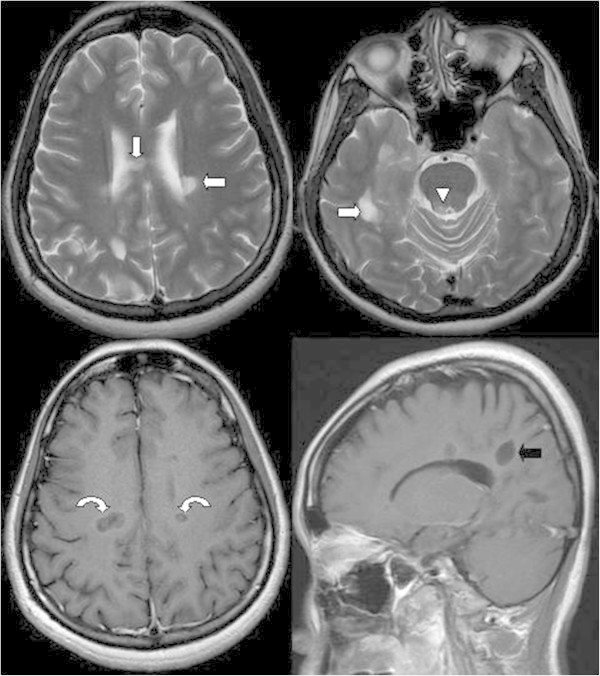
**Hyperintense lesions in the bilateral periventricular white matter (arrow), body of the corpus callosum (arrow), and periaqueductal grey matter (arrowhead) are noted on the T2-weighted image.** After gadolinium contrast injection, ring enhancement over these lesions is noted on the T1-weighted image (curved arrows), which is suggestive of active demyelination. In the sagittal view, multiple sclerosis plaques with a typical perpendicular orientation at the callososeptal interface are noted (Dawson’s fingers) (black arrow).

Because of these new symptoms, the patient underwent brain magnetic resonance imaging (MRI), and some hyperintense lesions in the bilateral periventricular white matter, body of corpus callosum, and periaqueductal grey matter (PAG) were noted on the T2-weighted images. After gadolinium contrast injection, ring enhancement over these lesions was noted on T1-weighted images. In the sagittal view, MS plaques with a typical perpendicular orientation at the callososeptal interface were noted (Dawson’s fingers) (Figure 1). The patient was diagnosed with definite MS according to the McDonald criteria (2010 revision). The MS diagnosis was based on the recording of one episode of MS attack and the evidence of simultaneous gadolinium-enhancing and non enhancing lesions. The patient refused to receive disease-modifying therapy because her symptoms were relieved and the headache subsided gradually.

**Figure 2:**
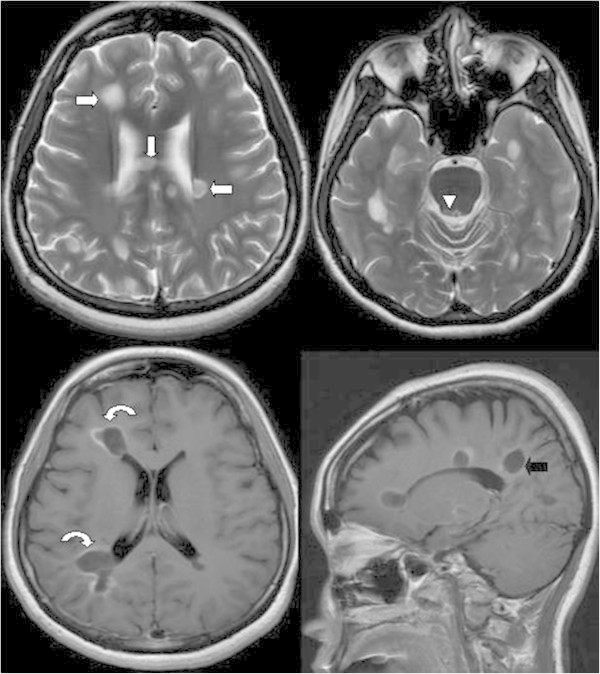
**Two months later, the hyperintense lesions located in the bilateral periventricular white matter (arrow), body of the corpus callosum (arrow), and right periaqueductal grey matter (arrowhead) were increased in number and size, as observed on T2-weighted images.** After gadolinium contrast injection, more prominent ring and incomplete ring enhancement over these lesions is noted on the T1-weighted image (curved arrows). In the sagittal view, multiple sclerosis plaques with an increased number of Dawson’s fingers are noted (black arrow).

**Figure 3:**
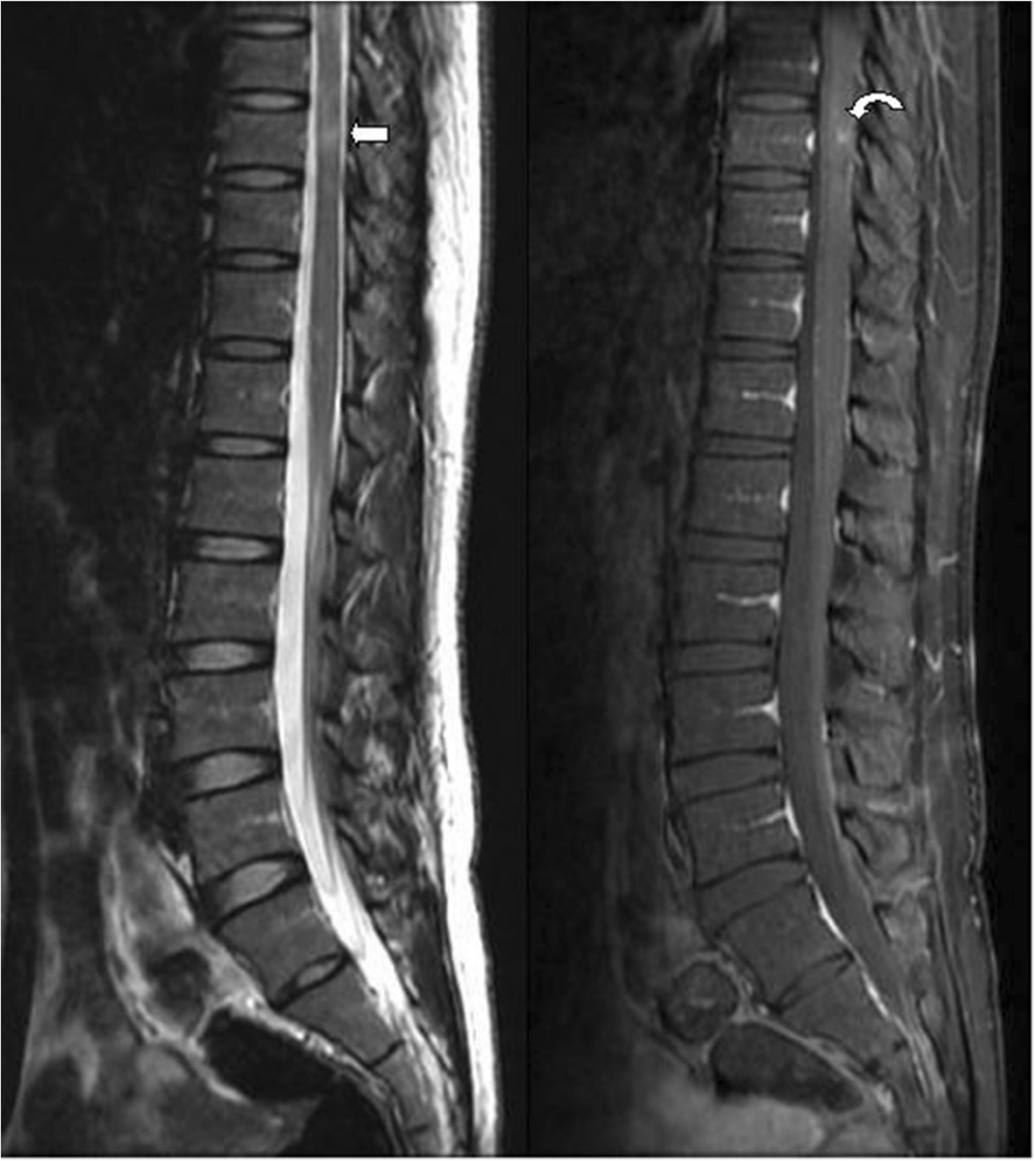
**Low thoracic and lumbar spine MRI showing a hyperintense lesion in the spinal cord (T9 level), which is observable on the T2-weighted image (arrow).** The lesion exhibits contrast enhancement after gadolinium injection (curved arrow).

Two months later, the patient was admitted to the neurology department of our hospital because of bilateral numbness of the lower legs, unsteady gait, difficulty in defecation, and urine retention for the past week. In addition, worsened migraine was noted again. Neurological examination showed bilateral pinprick-sensation and vibration impairment over the L1–L2 dermatome, positive Romberg test results, poor coordination, and short steps with wide-based gait. The follow-up brain MRI revealed that the above-mentioned lesions had increased in number and size, as observed on T2-weighted images. After gadolinium contrast injection, more prominent ring and incomplete ring enhancements over these lesions, including those in the PAG, were noted on T1-weighted images. In the sagittal view, MS plaques with an increased number of Dawson’s fingers were noted (Figure 2). These signs were suggestive of disease progression with active demyelination. On the basis of our physical observation, we performed a low thoracic and lumbar spine MRI, which showed a hyperintense lesion in the spinal cord (T9 level) on T2-weighted images and enhancement after gadolinium injection (Figure 3).

MS with spinal cord involvement was considered. The cerebrospinal fluid (CSF) analysis performed after admission to exclude other possible infectious causes showed no white cells and normal glucose (70 mg/dL) and protein (26 mg/dL) levels. CSF tests were negative for virus isolation and for Gram stain and culture. No oligoclonal bands were found. Serum analyses for presence of rheumatoid factor (RF), antibodies against nuclear antigen, and anticardiolipin immunoglobulins G and M yielded negative results; in addition, the levels of the C3 (90.7 mg/dL) and C4 (18.5 mg/dL) complement were normal. Furthermore, the anti-HIV and rapid plasma reagin (RPR) tests were negative. The erythrocyte sedimentation rate (ESR) and C-reactive protein (CRP) levels were <0.10 mg/dL. Assessment of visual evoked potentials (VEPs) revealed that the P100 wave latencies were 134 ms and 137 ms in the right and left eye, respectively; the amplitude was lower in the right eye. Compared to the normal values, the amplitudes were prolonged by over 20% in both the eyes, which indicated definite involvement of the optic nerves. After five days of methylprednisolone therapy, the clinical condition returned to the remission stage, without evident neurological deficit, and the patient’s headache improved significantly. After the second episode, we discussed the patient’s condition with her family and shifted the patient to immunomodulatory treatment with subcutaneous injection of 44 μg of interferon beta-1a thrice a week, with regular outpatient department follow-up.

## Discussion

Here, we report the case of a patient who showed worsening migraine as the initial presenting symptom of MS. MS was diagnosed according to the McDonald criteria (2010 revision). This patient had one episode of MS attack and exhibited the simultaneous presence of asymptomatic gadolinium-enhancing and non-enhancing lesions on the first brain MRI scan. The McDonald criteria for category III were established.

Patients with MS have a high prevalence of headache; headache occurs more commonly in patients with MS (27%) than in matched controls (12%) [7]. D’Amico and colleagues reported a 57.7% lifetime prevalence of headache in 137 patients with clinically definite MS [8]. However, it is unclear whether headache is a symptom of the onset of MS. Kurtzke et al. [9] considered headache as a subtle symptom of MS onset, with a variable frequency of 1.6–28.5%. This variation in frequency might be explained by differences in study design and patient inclusion criteria. Ophthalmoplegic migraine-like headache [10], complicated migraine [11], short-lasting unilateral neuralgiform headache attacks with conjunctival injection and tearing (SUNCT) [12], and cluster tic syndrome [13] have also been reported in single MS cases. Fragoso and Brooks [14] described a single case and Yetimalar et al. [15] reported the cases of two young women with migraine onset as the only symptom of the first episode of MS. These cases were different from our patient because they had no history of migraine without aura.

To our knowledge, there is no description in the literature of patients presenting with worsening migraine symptoms without neurological signs as the first episode of MS. When the initial symptoms of MS are worsening migraines and changes in headache patterns, they may be discounted as a recurrent event and ignored. Our study suggest that it is important to consider the possibility of MS in patients with worsening migraine symptoms accompanied by episodes of focal deficit and to follow-up these patients regularly.

Tortorella et al. found that patients with migraine had supratentorial lesions chiefly located in the frontal lobes and periventricular white matter and noted no difference in the number of supratentorial lesions between patients with migraine with or without aura [16]. In our case, the first brain MRI scan showed signs that were unlike the migraine lesions at the locations mentioned above, and there was damage to the periventricular and infratentorial PAG regions.

Many factors can trigger migraine attacks, such as changes in weather, drugs, alcohol, caffeine withdrawal, stress, fatigue, lack of sleep, hormonal therapy, diet, and hunger [17]. In our case, none of the above-mentioned factors was found. We hypothesize that stress causes inflammation in general, and particularly in MS, because stress-related neuropeptides activate the excretion of inflammatory molecules by microglia and mast cells [18]. Although no obvious stress was observed in our case, a hidden stress may have caused the acute MS attacks. As observed in our case, MS may be considered as one of the differential diagnoses of acute-migraine-like episodes.

The PAG modulates pain via the descending system and exerts an antinociceptive effect to the peripheral afferent system. Gee et al. showed that patients having MS with a plaque located within the PAG region displayed a four-fold increase in migraine-like headaches [19]. In our experience, common migraine attacks generally require no further imaging examinations. However, in our case, worsening migraine followed by an episode of focal neurological deficit indicated that we should look for any secondary causes of the symptoms. The involvement of the PAG region may explain why this case presented initially with worsening migraine headache without clinical response. The patient initially refused treatment when MS was diagnosed because of symptom relief; however, unfortunately, the symptoms recurred two months later. Although there is no cure for MS, the diagnosis of the disease and early initiation of an appropriate treatment are the best strategies to prevent severe neurological deficits.

Moreover, the more frequent administration of the IFNb preparation, as performed here, seems better than the administration of IFNb-1a just once a week [20,21]. In fact, in a previous study worsening of pre-existing headaches or de novo headaches were found only in patients with MS who received interferon therapy, and not in those who received other disease-modifying therapies [22]. Compared to natalizumab (NTZ), IFNb might cause a continued increase in the frequency and severity of migraine in cases of MS [23]. However, NTZ was approved by the US Food and Drug Administration as a second-line monotherapy for patients with MS who exhibit no response to other immunomodulating drugs [24]. In addition, glatiramer acetate is not available currently in our hospital. In our case, the patient receives a subcutaneous injection of interferon beta-1a thrice a week after well discussion with the patient.

## Conclusions

We demonstrated the importance of PAG involvement in a patient presenting with acute worsening migraine headache as an initial manifestation of MS. The presence of worsening migraine as a symptom of MS could lead to diagnostic difficulties. Moreover, this symptom may be easily ignored in patients with a migraine history. MS should be one of the differential diagnoses in young women showing a change in headache pattern or poor clinical drug response to headache treatment accompanied by episodes of focal neurological deficit. Failure to recognize MS may lead to inappropriate treatment and worse prognosis; therefore, early diagnosis in patients with MS is essential to improve their clinical outcomes and quality of life.

## Consent

Written informed consent was obtained from the patient for the publication of this report and accompanying images. A copy of the written consent is available for review by the Editor-in-Chief of this journal.

## Abbreviations

MS: Multiple sclerosis; CNS: Central nervous system; CIS: Clinically isolated syndrome; MRI: Magnetic resonance imaging; PAG: Periaqueductal gray matter; CSF: Cerebrospinal fluid; RF: Rheumatoid factor; RPR: Rapid plasma reagin; ESR: Erythrocyte sedimentation rate; CRP: C-reactive protein; VEP: Visual evoked potential; NTZ: Natalizumab.

## Competing interests

The authors declare that they have no competing interests.

## Authors’ contributions

GYL and CWW participated in the patient’s care, data collection, and manuscript writing. FCY participated in the patient’s care, data interpretation, and revision of the manuscript. All authors read and approved the final manuscript.
